# Understanding Public Perceptions of COVID-19 Contact Tracing Apps: Artificial Intelligence–Enabled Social Media Analysis

**DOI:** 10.2196/26618

**Published:** 2021-05-17

**Authors:** Kathrin Cresswell, Ahsen Tahir, Zakariya Sheikh, Zain Hussain, Andrés Domínguez Hernández, Ewen Harrison, Robin Williams, Aziz Sheikh, Amir Hussain

**Affiliations:** 1 Usher Institute The University of Edinburgh Edinburgh United Kingdom; 2 School of Computing Edinburgh Napier University Edinburgh United Kingdom; 3 University of Engineering and Technology Lahore Pakistan; 4 Edinburgh Medical School The University of Edinburgh Edinburgh United Kingdom; 5 Department of Computer Science University of Bristol Bristol United Kingdom; 6 Institute for the Study of Science, Technology and Innovation The University of Edinburgh Edinburgh United Kingdom

**Keywords:** artificial intelligence, sentiment analysis, COVID-19, contact tracing, social media, perception, app, exploratory, suitability, AI, Facebook, Twitter, United Kingdom, sentiment, attitude, infodemiology, infoveillance

## Abstract

**Background:**

The emergence of SARS-CoV-2 in late 2019 and its subsequent spread worldwide continues to be a global health crisis. Many governments consider contact tracing of citizens through apps installed on mobile phones as a key mechanism to contain the spread of SARS-CoV-2.

**Objective:**

In this study, we sought to explore the suitability of artificial intelligence (AI)–enabled social media analyses using Facebook and Twitter to understand public perceptions of COVID-19 contact tracing apps in the United Kingdom.

**Methods:**

We extracted and analyzed over 10,000 relevant social media posts across an 8-month period, from March 1 to October 31, 2020. We used an initial filter with COVID-19–related keywords, which were predefined as part of an open Twitter-based COVID-19 dataset. We then applied a second filter using contract tracing app–related keywords and a geographical filter. We developed and utilized a hybrid, rule-based ensemble model, combining state-of-the-art lexicon rule-based and deep learning–based approaches.

**Results:**

Overall, we observed 76% positive and 12% negative sentiments, with the majority of negative sentiments reported in the North of England. These sentiments varied over time, likely influenced by ongoing public debates around implementing app-based contact tracing by using a centralized model where data would be shared with the health service, compared with decentralized contact-tracing technology.

**Conclusions:**

Variations in sentiments corroborate with ongoing debates surrounding the information governance of health-related information. AI-enabled social media analysis of public attitudes in health care can help facilitate the implementation of effective public health campaigns.

## Introduction

SARS-CoV-2 is a major global threat to public health. Many governments worldwide are using mobile apps for contact tracing of citizens to contain the spread of SARS-CoV-2 [1]. The contact tracing applications used vary within and between countries, but they generally track movements of individuals and aggregate data to communicate to users when they may have been exposed to the virus, and if they need to be tested and/or self-isolated.

The higher the user base of contact tracing apps, the larger the anticipated impact on reducing the estimated effective reproduction number of the virus [2,3]. However, there is inconsistent and incomplete uptake of these technologies, especially in individualistic societies where intended users may perceive such apps to be of limited personal benefit or infringing on personal privacy and where the use of such apps is difficult to mandate [4,5]. To understand what public health measures may promote the use of contact tracing apps in the United Kingdom, policy makers need to understand the reasons why members of the public may be hesitant to use them. The empirical literature shows how attitudes of prospective users influenced by their individual backgrounds, technological features of the app in question, and perceived benefits and trade-offs can be a key barrier to the effective implementation of such apps (see Textbox 1) [6-9].

More than half of the world’s population, and around two-thirds of the UK population, currently use social media platforms, with significantly increased engagement levels during the ongoing pandemic [10]. We here sought to explore how analyzing public attitudes on these forums can provide insights into low levels of adoption of contact tracing apps in the United Kingdom. Our recent work has demonstrated the value of artificial intelligence (AI)–based sentiment analysis of social media data [11].

**Factors likely to impact the use of contact tracing apps identified in the literature.**

- **Individual backgrounds:** demographics, health status, involvement with COVID-19, and previous experiences (technology, health, and other factors associated with COVID-19).
- **Technological features:** effectiveness, privacy, security, cost, trust in system vendor, performance and reliability (eg, false-positive or false-negative alerts), compatibility with installed base, interoperability, and architecture of systems (eg, centralized vs decentralized).
- **Perceived benefits:** self, society, research, epidemiology, mediators (eg, if the user or others will take action based on risk, if they care about the environment or society, if they believe data will be used effectively), and tension between perceived benefit to the user and altruistic benefit to the whole population.
- **Perceived trade-offs, risks, and limiting factors:** data collected (who will know what about the user), risk of marginalization of certain demographic groups, level of control of who sees what aspects of data and who can retain it for how long, and transparency of app.

## Methods

### Data Sources

To assess the potential of an AI-based sentiment analysis to understand public views and concerns, we analyzed data from two popular social media platforms—Facebook and Twitter. Facebook posts were extracted using the CrowdTangle platform [12], and Twitter posts were extracted from the COVID-19 Twitter chatter dataset, constructed by Panacea Lab (using a publicly available Twitter application programming interface) [13]. English-language Facebook posts and tweets posted in the United Kingdom from March 1 to October 31, 2020, were extracted, and thematically filtered using a two-step process, for keywords related to both COVID-19 and contact tracing apps. The Twitter dataset was already filtered with predefined COVID-19–related keywords as detailed previously [13], and the same keywords were used to apply an initial filter to the Facebook data. The following contract tracing app–related keywords for the second step filtering were selected by our interdisciplinary team: “covid app,” “tracing app,” “contact tracing,” “privacy,” “security,” “app security,” “app privacy,” “contain virus spread,” “movement tracking,” “tracking,” and “surveillance.” Following geographical filtering for the United Kingdom, a total of 2000 tweets and 8000 Facebook posts were obtained for the analysis.

### Analysis

**Figure 1:**
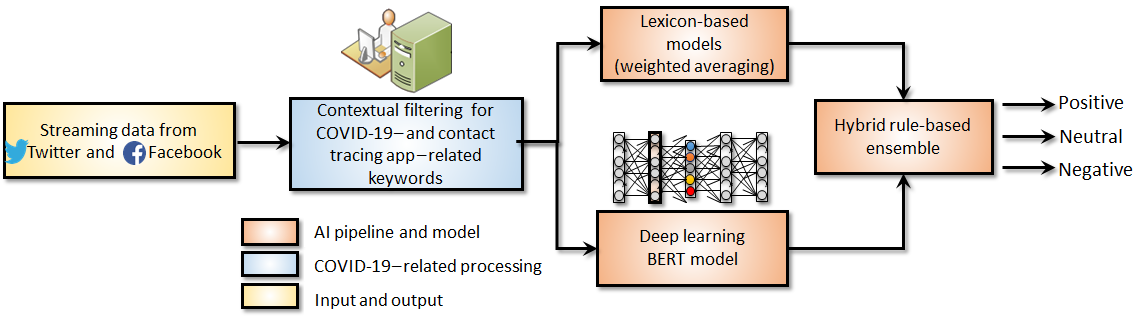
Hybrid rule-based ensemble model for sentiment classification of social media data. AI: artificial intelligence; BERT: Bidirectional Encoder Representations from Transformers.

A novel ensemble-based AI model developed by the authors in a recent study [14] was adapted and utilized for this study, by combining lexicon rule-based and deep learning (DL)–based approaches. Specifically, an average-weighting ensemble [15] of lexicon-based models, including Valence Aware Dictionary and Sentiment Reasoner (VADER) [16] and TextBlob [17], was combined with a state-of-the-art DL-based model Bidirectional Encoder Representations from Transformers (BERT) [18]. The overall hybrid rule-based ensemble method is illustrated in Figure 1.

The hybrid rule-based ensemble model was hyperparameter tuned based on manual validation by using 1000 randomly selected posts and labelled by the team. Specificity and sensitivity analyses showed that the lexicon-based model demonstrated better performance accuracy for positive sentiment and that the DL-based BERT model demonstrated enhanced accuracy for neutral and negative sentiments, as shown in the confusion matrices (Multimedia Appendix 1). The TextBlob lexicon-based model utilizes a higher weight (0.52) than the VADER model, as it demonstrated marginally better accuracy (VADER × 0.48 + TextBlob × 0.52) for positive sentiments. For the ensemble, weighted averaging was utilized to combine the two lexicon-based models. The output of the weighted average lexicons was combined with the DL-based BERT model through a rule-based approach. For positive sentiments, the weighted average output of the lexicon models was selected as the final output. For neutral and negative sentiments, the output of the DL-based BERT model was chosen as the final output. If-else logical constructs were used to determine the final sentiment class based on the base model outputs.

### Ethics

No ethical review was necessary since the data analyzed was fully in the public domain. A thorough assessment of the privacy risk to individuals posed by our research was conducted to ensure compliance with relevant sections of the General Data Protection Regulation (GDPR). We have also striven to comply with best practices for user protection to ensure no nonpublic material was included in our dataset.

### Data Availability

The code and data used for data analysis and generation of figures are openly available on GitLab [19] for reproducibility and transparency of the analysis. Due to the computationally expensive nature of the code, we recommend using a high-performance computing resource.

## Results

**Figure 2:**
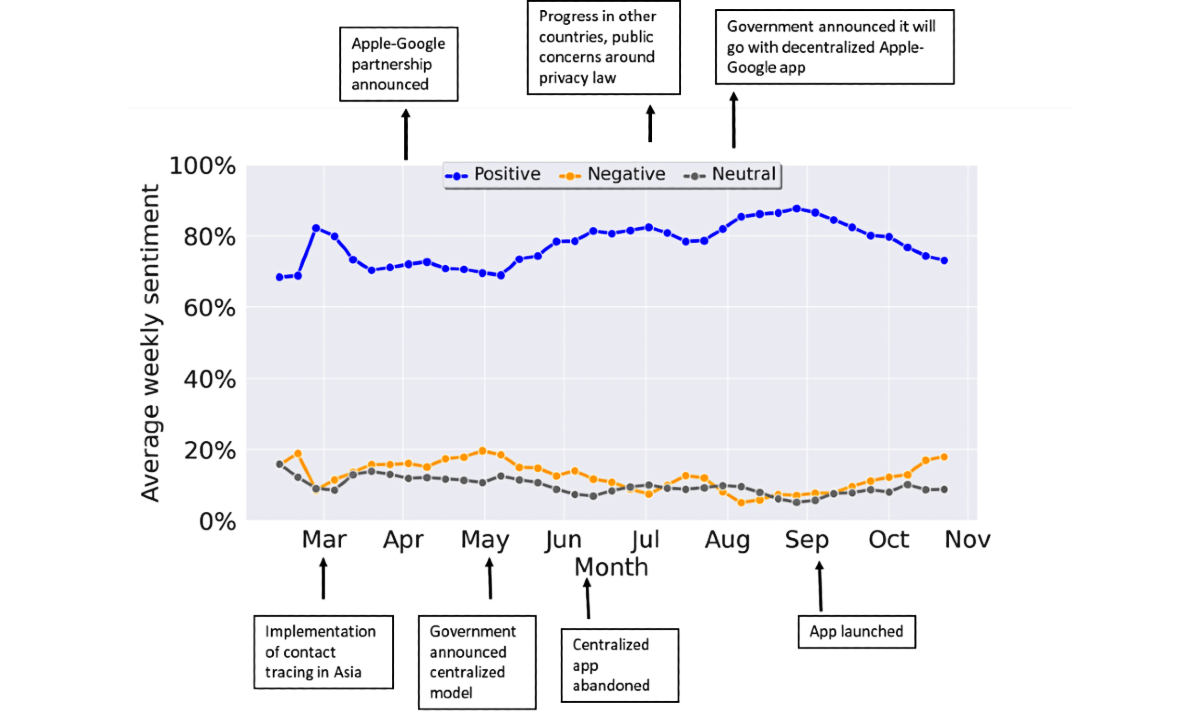
Average weekly sentiments extracted from Facebook and Twitter posts (combined) in the United Kingdom.

Figure 2 shows the average weekly sentiment trends on using contact tracing apps on Facebook and Twitter. The sentiments were computed by utilizing our ensemble-based AI model to predict the *overall* polarity of each post as positive, negative, or neutral. Due to the relatively small number, sentiments extracted from Facebook and Twitter posts were combined using weighted weekly averages. Sentiment word clouds were used to explore the topics being discussed at different time points of interest (Figure 2). Overall, the average positive sentiments were found to far outnumber the negative sentiments. We observed a six-fold difference between the sentiment type, with 76% positive and 12% negative sentiments. These were found to broadly corroborate with findings from independent surveys that show strong support for contact tracing apps. For example, an Ipsos MORI survey of 1983 UK adults conducted in May 2020 found that two-thirds (67%) of the population was in support and 12% was against the government’s plans to use a smartphone app to track and trace new cases of COVID-19 [20]. However, the public’s attitudes were found to be heavily influenced by demographics and the digital divide [12,21]. Several new survey studies are currently underway to explore public attitudes towards contact tracing apps.

Figure 2 illustrates some of the key events that are likely to have influenced changes in the public’s sentiments toward using contact tracing apps. Following the successful implementation of contact tracing strategies in several East Asian countries and high-profile research reporting that app-based contact tracing was likely to be effective in curbing the spread of the virus [22-24], Apple and Google partnered on developing a decentralized COVID-19 contact tracing technology [25]. Shortly after, the UK government released contact tracing guidance with plans to develop a *home-grown* app and deploy it through a centralized model, implying that individual information would be shared with health services [26,27]. This model was controversial, primarily driven by public concerns around privacy and security of the app, its incompatibility with iPhones, and potential breaches of the Data Protection Law [28]. In June 2020, the centralized UK app was abandoned [29]. In August 2020, the UK government decided to implement the decentralized contact tracing system developed by Apple and Google [30], launching the app in September 2020 [31]. Since then, there have been concerns around high rates of false-positive COVID-19 cases reported via the app, hindering its uptake among the public [32].

**Figure 3:**
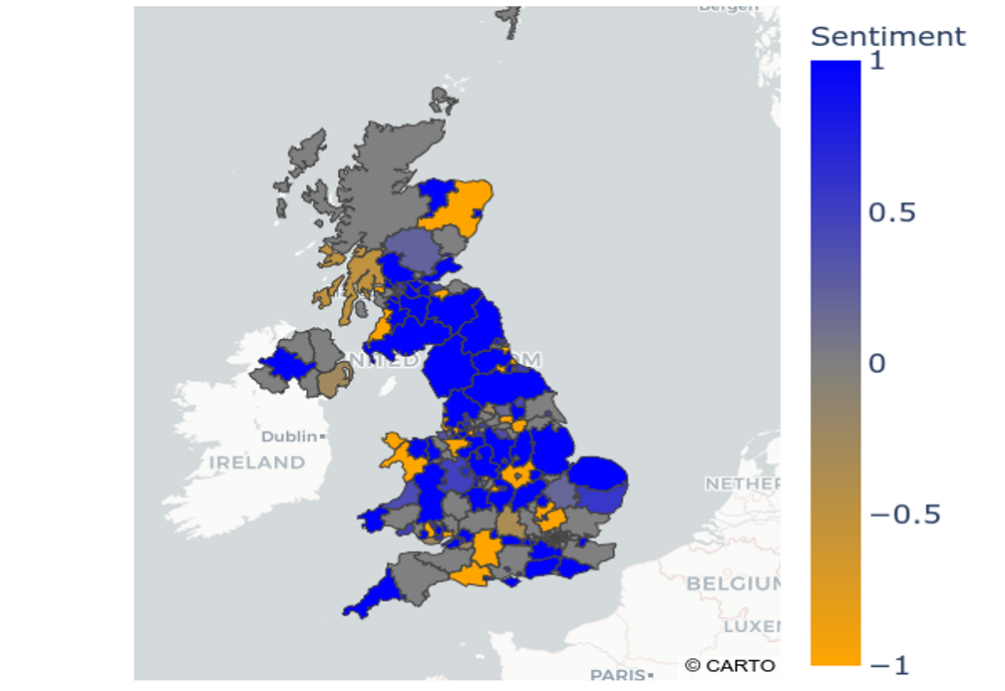
Spatial mapping of (averaged) UK public sentiment related to COVID-19 contact tracing apps on Twitter.

We also carried out spatial mapping of the sentiments extracted from geo-tagged tweets to counties in the United Kingdom (see Figure 3). Most tweets had an overall positive sentiment toward COVID-19 contact tracing. The counties with the most positive sentiment included Lincolnshire, Norfolk, Nottinghamshire, Leicestershire, and Northamptonshire in England, and Stirling, Fife, Dumfries and Galloway, East Ayrshire, and West Lothian in Scotland. Counties with the most negative sentiment were Suffolk, Somerset, Devon, and North Yorkshire—all in England.

## Discussion

### Principal Findings

This study has provided insights into some of the underlying issues and concerns surrounding contact tracing apps, and particularly how decisions surrounding system architecture (eg, centralized vs decentralized) can influence public attitudes.

More fine-grained spatiotemporal variation in sentiments across the United Kingdom, and globally, should be further studied. Failure to pay timely attention to negative attitudes and resistance to emerging technologies in population health can have potentially damaging consequences, whereas an understanding of barriers can facilitate the development of effective interventions.

Limitations of our preliminary analysis include that social media platform users are largely not representative of the total UK population (eg, users are younger, more left-wing, and have higher incomes) [33]. Only about 15% of UK adults are regular Twitter users, with activity levels varying among passive and active users, and the demographics of users do not reflect the demographics of the general population [34]. It has also been shown that sentiments of tweets can vary across geographical locations and may therefore reflect the demographics of the population being studied [35]. Variations in sentiments can occur at an individual level, influenced by personal experiences and changes not necessarily related to the subject of the tweet [36]. The developed ensemble-based sentiment analysis approach can be optimized by using additional labelled data for transfer learning and fine-tuning the BERT base model. Adaptive neuro-fuzzy inferencing [37] can also be utilized to replace the current nonadaptive (weighted-average and logical rule-based) constructs.

The current focus on selected social media platforms, outstanding issues around the accuracy of AI techniques (eg, around deceptive language), and the limited number of tweets and Facebook posts due to the specific search strategy used, as well as given that only geo-tagged tweets were used, may limit the generalizability of these findings. Thus, there is a need for a more refined and comprehensive search strategy using multiple social media and web platforms, as well as linked analysis with data from external trustworthy sources such as polls, census, surveys, and clinical notes. Considering the limitations, the proposed approach should currently only be used alongside other methods to assess public sentiment, including public consultations, surveys, and qualitative studies.

In future, mobility trends [38] can be integrated into this approach and demographic determinants can also be inferred [39] and included to provide further insights. Furthermore, subgroup analysis could explore reasons for low uptake in certain populations or communities (eg, anti-vaxxers [40]), which can inform the design of vaccine deployment strategies, including public messaging campaigns. The approach may help promote a learning public health policy cycle, where ideas can be tested against public attitudes before they are implemented, thereby maximizing their effectiveness and real-world applicability [41].

### Conclusions

We advocate for future work on AI-enabled social media analysis of public attitudes in health care, which we believe has the potential to help facilitate the implementation of effective public health campaigns. Through this preliminary analysis, we show how such innovative methods may complement findings using conventional methods to monitor public sentiments (eg, surveys) while also providing greater spatiotemporal granularity.

## Appendix

Multimedia Appendix 1 Normalized confusion matrices for VADER- and TextBlob-based lexicon models, deep learning-based BERT model, and the hybrid rule-based ensemble approach. BERT: Bidirectional Encoder-Representations from Transformers; VADER: Valence Aware Dictionary and Sentiment Reasoner.

## Abbreviations

AI: artificial intelligence
BERT: Bidirectional Encoder-Representations from Transformers
DL: deep learning
GDPR: General Data Protection Regulation
VADER: Valence Aware Dictionary and Sentiment Reasoner
