# Supplementary figures and images for: Understanding Public Perceptions of COVID-19 Contact Tracing Apps: Artificial Intelligence–Enabled Social Media Analysis
Source: J Med Internet Res. 2021 May 17;23(5):e26618. doi: 10.2196/26618 (PMC8130818; doi:10.2196/26618)

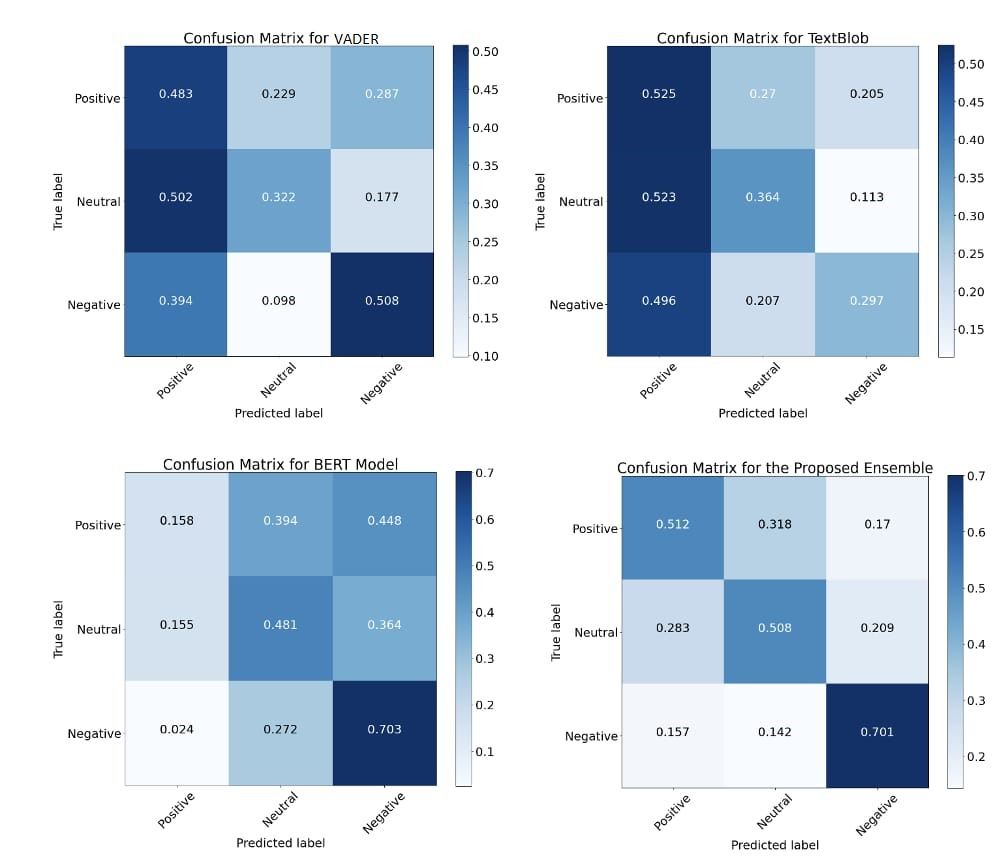

Supplement: Multimedia Appendix 1 [file jmir_v23i5e26618_fig.png]
